# A transposable element in a *NAC* gene is associated with drought tolerance in maize seedlings

**DOI:** 10.1038/ncomms9326

**Published:** 2015-09-21

**Authors:** Hude Mao, Hongwei Wang, Shengxue Liu, Zhigang Li, Xiaohong Yang, Jianbing Yan, Jiansheng Li, Lam-Son Phan Tran, Feng Qin

**Affiliations:** 1 Key Laboratory of Plant Molecular Physiology, Institute of Botany, Chinese Academy of Sciences, Beijing 100093, China; 2 Graduate University of the Chinese Academy of Sciences, Beijing 100049, China; 3 National Maize Improvement Center of China, China Agricultural University, Beijing 100193, China; 4 National Key Laboratory of Crop Genetic Improvement, Huazhong Agricultural University, Wuhan 430070, China; 5 Signaling Pathway Research Unit, RIKEN Center for Sustainable Resource Science, 1-7-22 Suehiro-cho, Tsurumi-ku, Yokohama 230-0045, Japan

## Abstract

Drought represents a major constraint on maize production worldwide. Understanding the genetic basis for natural variation in drought tolerance of maize may facilitate efforts to improve this trait in cultivated germplasm. Here, using a genome-wide association study, we show that a miniature inverted-repeat transposable element (MITE) inserted in the promoter of a *NAC* gene (*ZmNAC111*) is significantly associated with natural variation in maize drought tolerance. The 82-bp MITE represses *ZmNAC111* expression via RNA-directed DNA methylation and H3K9 dimethylation when heterologously expressed in *Arabidopsis*. Increasing *ZmNAC111* expression in transgenic maize enhances drought tolerance at the seedling stage, improves water-use efficiency and induces upregulation of drought-responsive genes under water stress. The MITE insertion in the *ZmNAC111* promoter appears to have occurred after maize domestication and spread among temperate germplasm. The identification of this MITE insertion provides insight into the genetic basis for natural variation in maize drought tolerance.

## Abstract (web summary)

Drought is a major cause of yield loss in maize and understanding the genetic determinants of natural variation in drought tolerance may aid breeding programs produce more tolerant varieties. Here, Mao *et al.* identify a MITE transposon insertion in a NAC transcription factor, which is associated with natural variation in drought tolerance.

## Graphical abstract

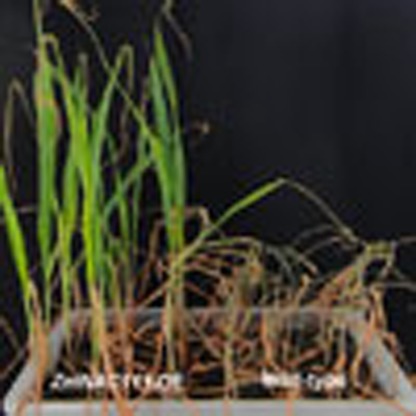

Maize (*Zea mays*) production is frequently compromised by water scarcity, which is aggravated by trends of climate warming and erratic rainfall patterns on a global scale^1^,^2^. During the past two decades, although progress has been made towards an overall increase in maize yields, plant sensitivity to drought stress has increased^1^. It is proposed that improving the ability of maize to overcome stress rather than primary productivity could be a primary contributor in efforts to achieve higher yielding maize^3^. Thus, enhanced drought tolerance has become a priority trait in current maize genetic improvement efforts. However, the identification of the genetic components underlying drought tolerance has proven to be challenging. To date, no quantitative trait loci (QTLs) responsible for maize drought tolerance have been cloned, despite the reports of their mapping information^4^,^5^,^6^,^7^.

Traditional QTL mapping, based on the genetic linkage of a certain trait with the molecular marker within a bi-parental segregation population, usually recombinant inbred lines (RILs), has been successfully used to identify the genes underlying QTLs for glume architecture^8^, branched architecture^9^, flowering time^10^, photoperiod sensitivity^11^, resistance to head smut^12^ and so on. However, it usually takes a relatively long time to generate RILs of the two parents, which are phenotypically contrasting with respect to the trait. Moreover, the mapping resolution is largely dependent on the genetic recombination among these RILs. To achieve accurate mapping and gene cloning, it usually requires either a large number of RILs initially or several consecutive steps of selfing or backcrossing, to narrow down the region containing the candidate gene. Thus, a large number of QTL mapping studies have been conducted but only a limited number of them were cloned^13^. Another limitation of this approach is that the functional variation(s) of only two parental alleles can typically be evaluated during the mapping process.

More recently, genome-wide association study (GWAS), which is based on genetic linkage disequilibrium (LD) and makes use of natural variation and recombinants, has been used as a novel strategy for dissecting complex trait loci in plants^14^,^15^,^16^,^17^. Since historical recombinant events can be exploited in a collection of a large number of genotypes, LD mapping can reach a high resolution and investigate multiple alleles of a single locus^14^. With the development of high-throughput DNA variation discovery technology and improvement of statistical analyses, GWAS has gained favourability in genetic research in various plant species. Especially, due to the rapid LD decay in the maize genome, GWAS has facilitated the genetic dissection of several complex traits, including kernel β-carotene^18^ and oil content^19^, and flowering time in maize^20^. Although association studies of maize drought tolerance have been attempted^21^,^22^,^23^,^24^, the proposed candidate genes or their causative variations still remain to be verified and resolved.

In the maize genome, ∼85% of the genomic contents are composed of transposable elements (TEs), and the generic sequences are embedded in a vast expanse of TEs^25^. To maintain stability of the genome, TEs are usually silenced and inactive, due to DNA and chromatin modifications^26^. However, TEs have been shown to play important roles in plant evolution and environmental adaptation. For instance, a hopscotch element inserted at ∼60-kb upstream of *teosinte branched1* (*tb1*) increased maize apical dominance^9^ and a *CACTA*-like TE located ∼2-kb upstream of *ZmCCT* was found to contribute to maize photoperiod sensitivity^20^. Miniature inverted-repeat transposable elements (MITEs) are a kind of non-autonomous DNA transposon, which are usually shorter than 600 bp and widespread in plant genomes^27^. A MITE insertion in ∼70-kb upstream of *ZmRAP2.7* was demonstrated to be associated with maize flowering time^10^. TEs can influence nearby gene expression either through the *cis*-acting element residing in their own sequences, or by changing the DNA or chromatin methylation status of adjacent genes^26^,^28^,^29^. In rice (*Oryza sativa*), MITEs have been recently discovered to be capable of generating 24-nt small interfering RNA (siRNA), depending on Dicer-like 3a (OsDCL3a) activity, and interfering nearby gene expression through RNA-directed DNA methylation (RdDM)^30^. In plants, the RdDM pathway consists of the following major steps: (1) the RNA polymerase IV transcribes single-strand RNAs from repetitive heterochromatin regions; (2) its physically associated RNA-dependent RNA polymerase 2 (RDR2) synthesizes the double-stranded RNA (dsRNAs); (3) the dsRNAs are cleaved by DCL3 into 24-nt siRNAs; and (4) ARGONAUTE 4 (AGO4) subsequently loads the siRNAs to their complementary DNA regions. Lastly, the formed complex recruits the DNA methyltransferase (DRM2) to catalyse methylation at cytosine in CG, CHG and CHH contents (H=A, G or C), especially in CHH sequence context, which is a hallmark of RdDM^31^,^32^,^33^. Enrichment of chromatin histone3 lysine 9 dimethylation (H3K9me2), which is mainly catalysed by a histone methyltransferase, SUVH4 (also named KYP), couples the DNA methylation in the adjacent regions^30^,^34^.

Here, we show by GWAS that an 82-bp (MITE) insertion in the promoter region of a *NAC* gene (*ZmNAC111*) is associated with maize drought tolerance. The MITE insertion correlates with lower *ZmNAC111* expression in maize, and when heterologously expressed in *Arabidopsis* it suppresses *ZmNAC111* expression via the RdDM pathway. Transgenic studies demonstrated that enhanced expression of *ZmNAC111* conferred drought tolerance in both transgenic *Arabidopsis* and maize seedlings, improves plant water-use efficiency (WUE) and enhances the expression of stress-responsive genes under the stress. A comparison of MITE insertion frequency and nucleotide diversity at the *ZmNAC111* locus among teosinte, tropical/subtropical (TST) and temperate genotypes suggests that the MITE insertion appears to have occurred after maize domestication from teosinte and spread in the temperate germplasm.

## Results

### *ZmNAC111* is associated with maize seedling drought tolerance

To identify genes associated with maize drought tolerance, we performed GWAS by analysing a natural maize population, consisting of 368 inbred lines that were collected from TST and temperate regions of the world^19^. Approximately, 560,000 single nucleotide polymorphisms (SNPs)^19^ were applied to the study. Considering the complexity of plant drought tolerance, which is affected by both the time period and intensity of the stress imposed to plants, we decided to focus on tolerance to severe drought stress at the seedling stage. Seedling survival rate (SR) can reflect plant tolerance mechanisms and cellular responses to drought. It is less affected by environmental fluctuation, which helps to identify of the underlying genetic determinant(s). The drought tolerance of each genotype was assayed by calculating an SR index (percentage of the survived plants after re-watering) under severe drought stress at seedling stage^24^. On average, the inbred lines from TST exhibited higher SR in comparison with those from temperate regions, indicating that maize germplasm derived from areas near to the place of origin may be more drought tolerant than those cultivated in temperate regions. On the whole-genome scale, in consideration of the population structure (Q) and parental relatedness (K) of the population, a SNP within *GRMZM2G127379* on chromosome 10 was identified to be significantly associated with plant drought tolerance (Supplementary Fig. 1). *GRMZM2G127379* encodes a NAC-type transcription factor (TF), belonging to a family with more than 100 members in maize genome. *GRMZM2G127379* has previously been designated as *ZmNAC111* (ref. 35). Phylogenetic analysis of the amino acid sequence encoded by *ZmNAC111* indicated that its closest identified homologous gene in rice is *OsNAC10* (*LOC_Os11g03300*)^36^ (Supplementary Fig. 2).

**Figure 1:**
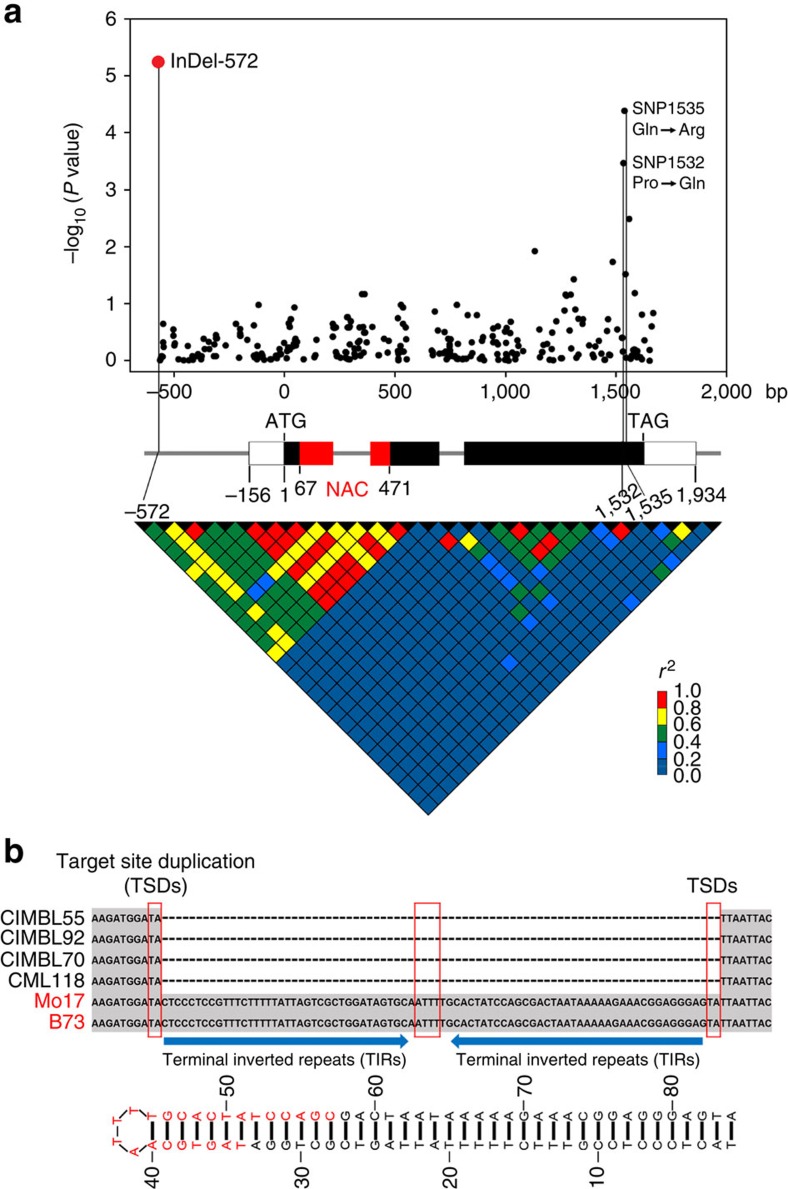
An 82-bp MITE insertion in the *ZmNAC111* promoter associated with maize drought tolerance. (**a**) The association analysis of genetic variation in *ZmNAC111* with drought tolerance in maize and the pattern of pairwise LD of DNA polymorphisms. A schematic diagram of the 2.3-kb genomic region of *ZmNAC111*, including the 5′-, 3′-UTR, three exons and two introns is presented. The location of the start codon (ATG) is labelled as ‘+1'. The region encoding the NAC domain is indicated in red. The most significant InDel (InDel-572) in the promoter and two non-synonymous variations in the coding region are connected to their locations in the gene diagram by solid lines. SNP1532 produced a change of Pro to Gln in the encoded protein, and SNP1535 changed Gln to Arg. (**b**) The DNA sequence and structure of the 82-bp MITE inserted in the *ZmNAC111* promoter. The target site duplications (TSDs) and the loop are indicated by the red boxes. The blue arrows indicate two terminal inverted repeats. The predicted hairpin structure of the MITE is illustrated at the bottom. A 24-nt siRNA sequence found in the Cereal Small RNA Database that aligned to the MITE is highlighted in red. The MITE is present in the promoter of the *ZmNAC111* gene in many of the drought-sensitive genotypes, such as B73 and Mo17, whereas it is absent in drought-tolerant genotypes, such as CIMBL55, 92, 70 and CML118.

NAC proteins regulate multiple biological processes in plants, including cotyledon^37^ and root development^38^, formation of secondary walls^39^, leaf senescence^40^, nutrient remobilization to grains^41^ and stress responses^42^. Considering the possible function of *NAC*-type genes in plant drought tolerance, we then sequenced the *ZmNAC111* gene in 262 maize inbred lines. A 2.3-kb genomic region, spanning the 5′- to 3′-untranslation region (UTR) of *ZmNAC111*, was analysed. A total of 157 SNPs and 119 InDels (insertions and deletions) were further identified. A newly identified 82-bp InDel (InDel-572), located 572-bp upstream of the start codon of *ZmNAC111*, was found to have the greatest significant association with the seedling SR (*P*=5.52 × 10^−6^; calculated under mixed linear model, see Methods; Fig. 1a), contributing to 7.27% of the phenotypic variation in the natural population. Two non-synonymous variations in exon3 were also identified as marginally significant (Supplementary Data 1). SNP1532 resulted in an amino acid residue change of proline (Pro) into glutamine (Gln), and SNP1535 resulted in an alteration of Gln to arginine (Arg). Both mutations, which were in strong LD, locate in the C-terminal transcriptional regulatory region but not in the N-terminal DNA-binding domain of the ZmNAC111 protein. InDel-572 was in LD with the variations in 5′-UTR and the first exon (*r*^2^>0.4), but not with the two non-synonymous SNPs (Fig. 1a). All the other variations were not associated to the trait with statistical significance.

### InDel-572 is a MITE insertion in the *ZmNAC111* promoter

Sequence analysis of InDel-572 in the promoter region of *ZmNAC111* gene revealed that it is composed of a long-terminal inverted repeat (38-bp for each), a 4-bp loop and two additional nucleotides ‘TA' at the end. Another ‘TA' sequence was found directly before this insertion (Fig. 1b). It represents a typical structure of a MITE insertion within the genome, which is usually short with approximately hundreds of bps and consists of TIRs and target-site direct repeats, with a preferential insertion at TA or TAA^27^. We blasted the maize TE database using the 82-bp sequence as a query (http://maizetedb.org/~maize/) and found that it belongs to the *Tc1/Mariner* superfamily of MITEs. The 80-bp DNA sequence, excluding the target-site direct repeat, can form a perfect stem-loop structure (Fig. 1b). It is present in the promoter of *ZmNAC111* of drought-sensitive genotypes, such as B73 and Mo17, whereas it is absent in the drought-tolerant genotypes, such as CIMBL55, 92, 70 and CML118 (Fig. 1b).

### The MITE insertion correlates with lower *ZmNAC111* expression

**Figure 2:**
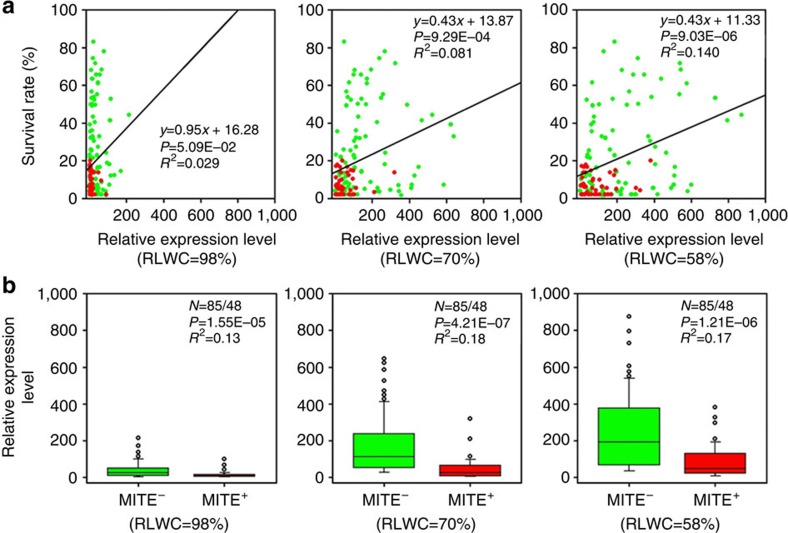
Expression level of *ZmNAC111*. Relative expression level of *ZmNAC111* under well-watered, moderate and severe drought conditions in relation to the rate of plant survival, and the presence or absence of the MITE (InDel-572) insertion. (**a**) Correlation of plant survival rate with the relative expression level of *ZmNAC111*. Drought stress was estimated by the decrease in the relative leaf water content (RLWC) from 98% (well watered) to 70% (moderate drought) and to 58% (severe drought). The red and green dots represent genotypes with or without the MITE insertion (MITE^+^ and MITE^−^) in the *ZmNAC111* promoter, respectively. Significance is determined using the *t*-test. (**b**) Comparison of *ZmNAC111* expression in MITE^−^ and MITE^+^ genotypes in relation to RLWC. One-way analysis of variance (ANOVA) is applied to determine statistical differences in *ZmNAC111* expression.

Since the MITE insertion locates in the promoter region of *ZmNAC111*, we hypothesized that it results in altered *ZmNAC111* expression among different genotypes. To examine this hypothesis, we analysed the expression of the *ZmNAC111* gene among 133 inbred lines under well-watered, moderate and severe drought conditions. Quantitative reverse-transcription PCR (qRT–PCR) analysis of a total of 399 RNA samples revealed that *ZmNAC111* expression positively correlated with plant SR under both moderate and severe drought stresses, but not under well-watered conditions (Fig. 2a; Supplementary Data 2). This finding suggested that increased expression of *ZmNAC111* might contribute to the drought tolerance of natural maize varieties examined in this study under water stress. Furthermore, the qRT–PCR result revealed that regardless of the levels of stress imposed, the genotypes without the MITE (MITE^−^) had significantly higher expression of *ZmNAC111* than those with the MITE insertion (MITE^+^; Fig. 2b). The contrasting phenotype of drought tolerance and the differential *ZmNAC111* expression of several representative genotypes were shown (Supplementary Fig. 3). On the basis of these data, we suggest that the MITE insertion may repress *ZmNAC111* expression resulting in higher sensitivity of MITE^+^ maize varieties to drought stress.

To further test the association between the MITE insertion at the *ZmNAC111* locus and seedling drought tolerance, we constructed three bi-parental F_2:3_ populations. The genotypes of two parents of each population were either MITE^+^ or MITE^−^ at the *ZmNAC111* locus. We observed that the MITE^−^ allele co-segregated with drought tolerance in three bi-parental F_2:3_ populations (Supplementary Fig. 4). On the other hand, when we compared the transactivation activity of ZmNAC111 proteins encoded by alleles that differed in the two non-synonymous variations, SNP1532 and SNP1535, we found that these two variations in different alleles did not significantly affect the transactivation activity of the ZmNAC111 (Supplementary Fig. 5). Collectively, on the basis of our results, we suggest that the MITE insertion is the likely cause for differences in drought tolerance associated with *ZmNAC111*, rather than amino acid changes in the encoded protein, and that the MITE^+^ is the drought-sensitive and MITE^−^ is the drought-tolerant allele of *ZmNAC111*.

### Correlation with DNA and histone methylation differences

**Figure 3:**
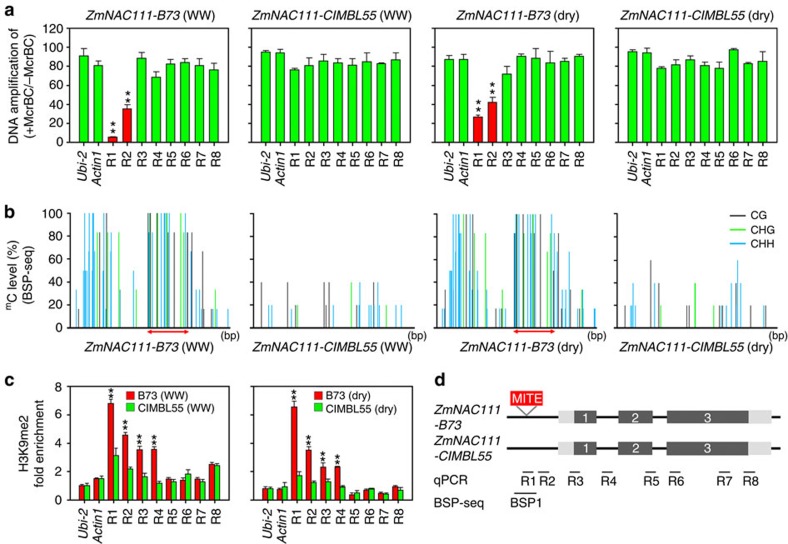
DNA and H3K9me2 methylation status of the drought-tolerant and drought-sensitive alleles of *ZmNAC111*. (**a**) DNA methylation status was determined by treatment with McrBC, a methylation-sensitive endonuclease followed by qPCR (McrBC–qPCR) analyses in the eight regions (R1–R8) of the genomic sequence of *ZmNAC111*. Genomic DNA was extracted from B73 and CIMBL55 genotypes grown under well-watered (WW) and moderate drought (dry, RLWC=70%) conditions. (**b**) Methylation of cytosine residues in CG, CHG and CHH sites (grey, green and blue lines, respectively) was revealed by bisulphite sequencing of the BSP1 region. The DNA samples of (**a**) were analysed. The MITE region is indicated by a double-sided arrow. (**c**) Chromatin state was detected using an anti-H3K9me2 ChIP–qPCR assay at eight different regions (R1–R8) of the genomic regions of *ZmNAC111-B73* and *ZmNAC111-CIMBL55*. The H3K9me2 state of the maize *Ubi2* and *Actin1* were tested in parallel as negative controls. Anti-H3 was used as an internal reference in the ChIP–qPCR assay. (**d**) The positions of R1–R8 in the genomic region of *ZmNAC111*. The 5′- and 3′-UTR regions (light grey boxes), exons (grey boxes) and 82-bp MITE insertion (red box) are illustrated. Black lines indicate the position of the McrBC–qPCR, ChIP–qPCR and bisulphite sequencing (BSP1) analyses. Error bars are s.d. and significant differences are determined using the *t*-test, **P<0.01.

To understand why the 82-bp MITE insertion may be correlated with reduced *ZmNAC111* expression, we checked the DNA and histone methylation status of *ZmNAC111* in the MITE^−^ and MITE^+^ genotypes. Eight regions (R1–R8) spanning the *ZmNAC111* gene and promoter in two inbred lines, B73 (drought sensitive, MITE^+^) and CIMBL55 (drought tolerant, MITE^−^), were representatively analysed. Results revealed that only R1 and R2, nearest the MITE insertion, were hypermethylated in *ZmNAC111-B73* but not in *ZmNAC111-CIMBL55*, regardless of the stress treatment (Fig. 3a). Bisulphite sequencing of these regions detected DNA hypermethylation, especially at the CHH content (Fig. 3b). Moreover, chromatin immunoprecipitation (ChIP) using a specific H3K9me2 antibody and followed by qPCR analysis demonstrated that H3K9me2 was significantly enriched in R1–R4 in *ZmNAC111-B73* in comparison with that in *ZmNAC111-CIMBL55*. The H3K9me2 levels of the other regions, however, remained comparable. On the basis of these data, we suggest that the MITE insertion represses *ZmNAC111* expression through DNA and histone methylation of its nearby regions (Fig. 3c,d).

### Repression of *ZmNAC111* expression through RdDM pathway

**Figure 4:**
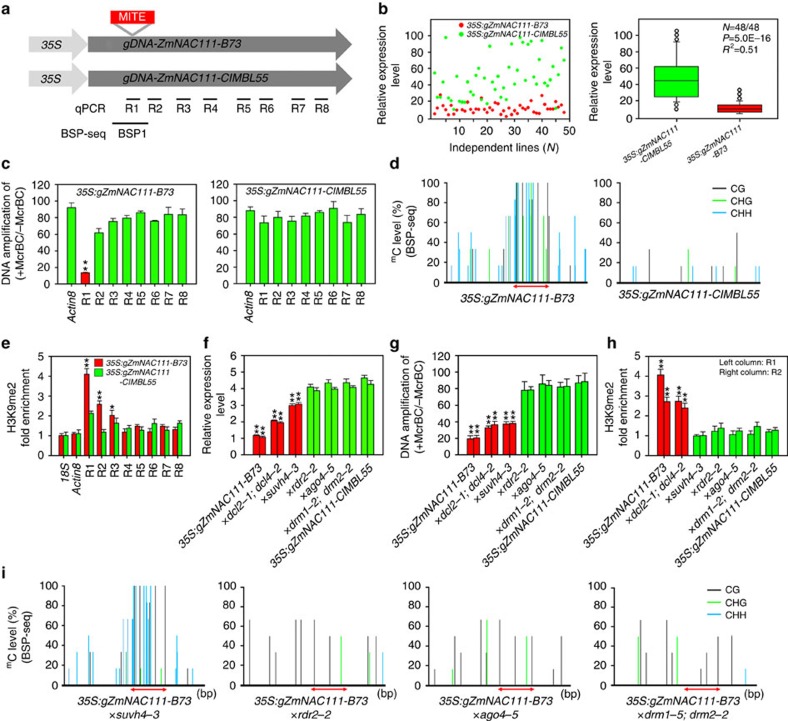
Repression of *ZmNAC111* expression by the MITE insertion is dependent on RNA-directed DNA methylation and histone methylation when heterologously expressed. (**a**) Diagram of the plasmid constructs (*35S:gZmNAC111-B73* and *35S:gZmNAC111-CIMBL55*) that were used to transform *Arabidopsis*. The MITE insertion (red box), regions of the McrBC–qPCR, ChIP–qPCR (R1–R8) and bisulphite sequencing (BSP1) analyses are indicated. (**b**) Left panel: comparison of *ZmNAC111* expression in independent transgenic lines of *35S:gZmNAC111-B73* and *35S:gZmNAC111-CIMBL55*. Right panel: statistical differences in the *ZmNAC111* gene expression in the transgenic *Arabidopsis* lines. (**c**) DNA methylation status in the eight regions (R1–R8) of the genomic sequence of *35S:gZmNAC111-B73* and *35S:gZmNAC111-CIMBL55* determined by McrBC–qPCR assays. (**d**) Methylation states of cytosine residues in CG, CHG and CHH sites, assayed with bisulphite sequencing of the BSP1 region. (**e**) Chromatin states detected at the eight regions (R1–R8), using an anti-H3K9me2 ChIP–qPCR assay. The H3K9me2 states of the *18S* and *Actin8 Arabidopsis* genes were evaluated in parallel and served as negative controls. Anti-H3 was used as an internal reference for ChIP–qPCR. (**f**) qRT–PCR analysis of transcript levels of *35S:gZmNAC111-B73* in wild-type and the RdDM mutants, and *35S:*g*ZmNAC111-CIMBL55* transcript levels in the wild type. (**g**) DNA methylation status determined using the McrBC–qPCR assay of the R1 region in the designated genetic backgrounds. (**h**) Chromatin states detected using anti-H3K9me2 ChIP–qPCR assays at R1 (left column) and R2 (right column) region. ‘* × dcl2-1;dcl4-2*'; ‘* × suvh4-3*'; ‘* × rdr2-2*'; ‘* × ago4-5*'; and ‘ × *drm1-2;drm2-2*' in (**f**) and (**g**) indicate the homozygous genetic background of the *35S:gZmNAC111-B73-2* (left column) and *-23* (right column) transgenics after crossing. Green columns indicate that *ZmNAC111* expression (**f**), DNA methylation (**g**) and H3K9me2 (**h**) were comparable with levels in the *35S:gZmNAC111-CIMBL55* transgenics in the wild-type background, whereas red columns indicate that they are significantly different with those in the *35S:gZmNAC111-CIMBL55* transgenics. (**i**) Methylation of cytosine residues assayed with bisulphite sequencing of the BSP1 region of the *35S:gZmNAC111-B73* transgenics in the different RdDM-defective mutant backgrounds. The MITE region is indicated by double-sided arrows. Error bars are s.d. and significant differences are determined using the *t*-test, *P<0.05; **P<0.01.

A considerable number of 21- to 24-nt siRNA species were identified that could be aligned to the MITE sequence in the maize small RNA database (http://sundarlab.ucdavis.edu/smrnas/; Supplementary Fig. 6). In an attempt to check the hypothesis that the 82-bp MITE insertion may mediate DNA and histone methylation through the RdDM pathway, the genomic fragments of *gZmNAC111-B73* and *gZmNAC111-CIMBL55* were transformed into *Arabidopsis* and expressed with the *CaMV 35S* promoter (Fig. 4a). The resultant 48 independent T_2_ transgenic lines for each construct were analysed. The transgenic plants harbouring *35S:gZmNAC111-CIMBL55* generally exhibited significantly higher levels of *ZmNAC111* expression, compared with the *35S*:*gZmNAC111-B73* transgenics (Fig. 4b). DNA hypermethylation and H3K9me2 enrichment in the region nearby the MITE insertion were expectedly observed in the *35S:gZmNAC111-B73*, but not in *35S:gZmNAC111-CIMBL55* transgenic lines (Fig. 4c–e). Plant drought tolerance was then compared between these two types of transgenics. The transgenic *Arabidopsis* harbouring *35S:gZmNAC111-CIMBL55* displayed greater drought tolerance than those transformed with *35S:gZmNAC111-B73* (Supplementary Fig. 7).

To verify that the MITE insertion-mediated *ZmNAC111* repression through the RdDM pathway when heterologously expressed in *Arabidopsis*, five mutants defective in key genes involved in the RdDM pathway were crossed with two independent homozygous *35S:gZmNAC111-B73-2* and *-23* lines to transfer the *35S:gZmNAC111-B73* construct to the RdDM-defective backgrounds. In the *drm1-2;drm2-2*, *ago4-5* and *rdr2-2* mutants, *ZmNAC111-B73* expression was greatly enhanced, while its DNA methylation and H3K9me2 were reduced to a comparable level relative to those of the *ZmNAC111-CIMBL55* in the wild-type background. In the *suvh4-3* mutant, the H3K9me2 level of *ZmNAC111-B73* was significantly reduced, but the DNA hypermethylation remained relatively similar to that in the wild-type background, which was consistent with the fact that the SUVH4 functions as a histone methyltransferase. In *dcl2-1;dcl4-2*, the gene expression, DNA methylation and H3K9me2 levels of *ZmNAC111-B73* still differed from those observed for the *ZmNAC111-CIMBL55* in wild-type background, most likely due to the intact *DCL3* function in this mutant (Fig. 4f–i). No remarkable change in gene expression, DNA methylation and H3K9me2 level was observed when *35S:gZmNAC111-CIMBL55-5* and *-12* transgenic *Arabidopsis* plants were crossed with the RdDM mutants (Supplementary Fig. 8). Collectively, these data clearly indicated that MITE represses the *ZmNAC111* expression through the RdDM pathway when heterologously expressed in *Arabidopsis*.

### *ZmNAC111* overexpression confers seedling drought tolerance

Given that *ZmNAC111* expression is positively correlated with maize drought tolerance, we generated both transgenic *Arabidopsis* and maize, overexpressing the coding sequence of *ZmNAC111* (from the B73 genotype). For the transgenic *Arabidopsis*, the phenotypes of three independent *35S:ZmNAC111* lines were analysed. In comparison with the empty-vector transformed plants (VC), the transgenic *Arabidopsis* displayed significantly enhanced drought tolerance, without remarkable morphological changes under normal growth conditions. When the survival of VC was around 20%, ∼80% of the transgenic plants were alive in the parallel water-withholding experiments (Supplementary Fig. 9a–c). The transgenic *Arabidopsis* plants were also hypersensitive to exogenous abscisic acid (ABA) as shown by seed germination and stomatal closure assays, indicating an enhancement of ABA signalling in the transgenic plants (Supplementary Fig. 9e–g).

**Figure 5:**
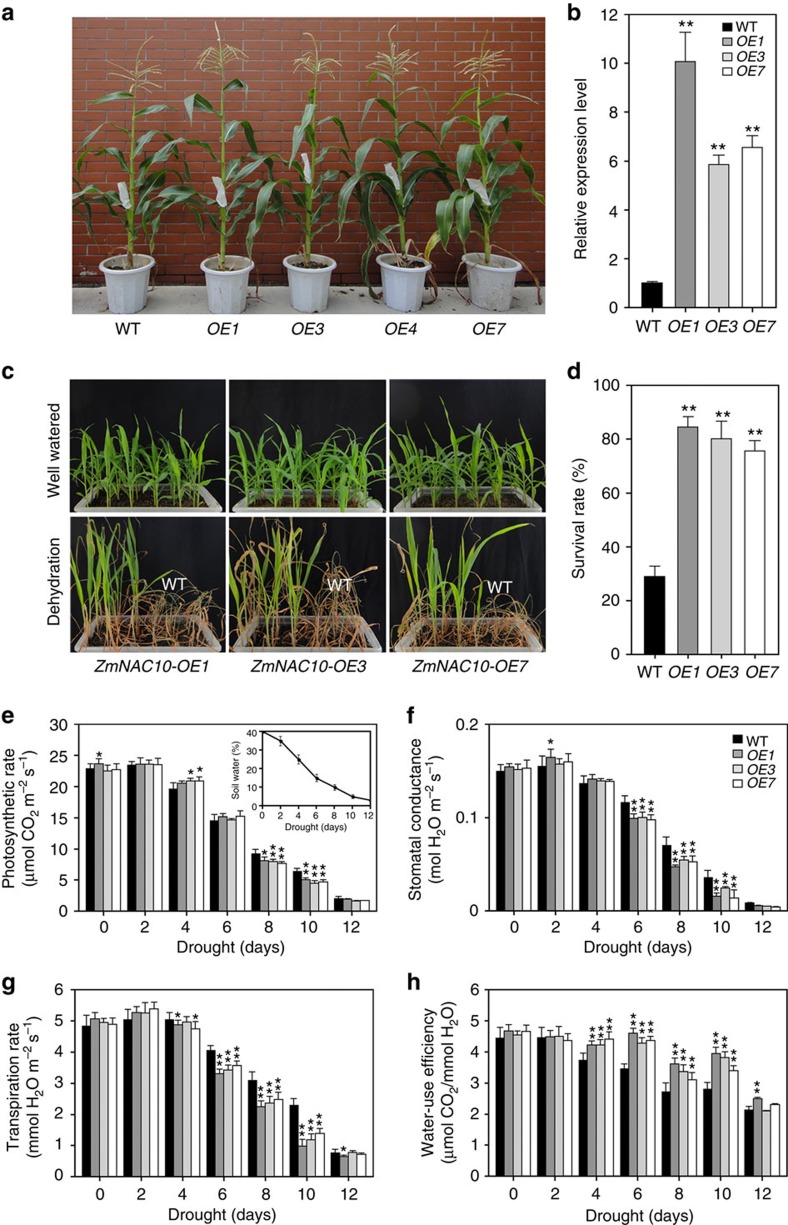
Drought tolerance of *ZmUbi:ZmNAC111* transgenic maize. (**a**) The growth phenotype of the T_1_ generation transgenic and sibling transformation-negative (WT) plants. Four representative independent transgene-positive lines (*ZmNAC111-OE1*, *OE3*, *OE4* and *OE7*) and the WT are shown. (**b**) Transcript levels of *ZmNAC111* in the WT, and three independent *ZmUbi:ZmNAC111* transgenic maize lines. (**c**) Drought tolerance of T_2_ seedlings of *ZmUbi:ZmNAC111* transgenic maize compared with WT. Photographs were taken under well-watered conditions and subsequent to a drought treatment followed by re-watering for a period of 7 days. The survival rates of WT and transgenic *ZmUbi:ZmNAC111-OE1*, *OE3* and *OE7* plants were compared. (**d**) Statistical analysis of survival rates after drought treatment and recovery. The average percentage of survival and standard errors were calculated from four independent experiments. (**e**–**h**) Comparison of the photosynthetic performance of *ZmUbi:ZmNAC111* transgenic and WT plants during the process of the drought stress. (**e**) Photosynthesis rate; (**f**) stomatal conductance; (**g**) transpiration rate; and (**h**) water-use efficiency. Error bars are s.d. and significant differences are determined using the *t*-test, *P<0.05; **P<0.01.

Similar improved drought tolerance was also observed in pot-grown transgenic maize transformed by *ZmUbi:ZmNAC111.* Under drought stress, ∼80% of the T_2_ generation transgenic maize plants survived; whereas, the SR of the transgenic-negative sibling plants (WT) was only 30% (Fig. 5c,d). No evident abnormal changes were observed in the transgenic maize compared with WT under normal growth conditions, although we acknowledge that these growth conditions likely do not accurately represent field conditions (Fig. 5a,b; Supplementary Data 3). Next, we compared the stomatal response and transpiration under progressive water stress between the transgenic maize and WT. The leaf photosynthesis rates (PS), stomatal conductance (SC) and transpiration rates (TR) were recorded every other day for 12 days in both WT and transgenic maize, when the soil water content (SWC) decreased from 40% to near 2%. During the first 2 days (SWC>30%), the three physiological parameters were comparable between the transgenics and WT, supporting the observation that they did not differ in growth under unstressed conditions (Fig. 5e–g). When SWC decreased to about 20%, TR of the transgenics was initially measured to decrease, resulting in a significantly greater WUE (calculated as PS in relation to TR) of the transgenic maize plants relative to WT (Fig. 5g,h). With the SWC dropping to ∼15%, the SC and TR became significantly smaller in the transgenics than those in WT, whereas, the PS remained comparable (Fig. 5e). Thus, WUE of the transgenic maize was maintained greater than that of the WT (Fig. 5g). Afterwards, the PS of the transgenics was more reduced compared with that of WT; however, due to a more remarkably reduced SC and TR, the WUE of the transgenics still remained significantly greater than that of WT, until SWC dropped to ∼2% (Fig. 5h). These data revealed that the transgenic maize plants had a greater WUE under water deficit in comparison with WT, which likely conferred enhanced drought tolerance to the transgenic maize.

**Figure 6:**
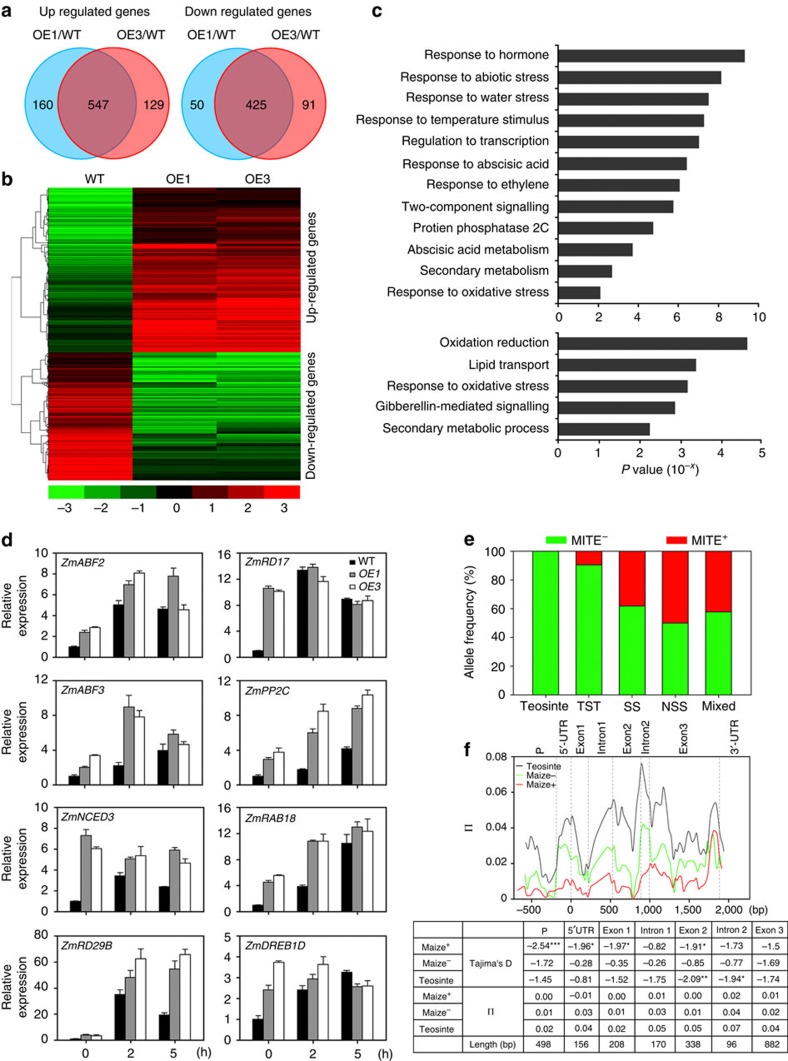
Transcriptomic analysis of *ZmUbi:ZmNAC111* transgenic maize and comparison of MITE insertion frequency and nucleotide diversity of ZmNAC111 in teosinte, TST and temperate maize inbred lines. (**a**) Venn diagrams of upregulated or downregulated genes in *ZmUbi:ZmNAC111-OE1* and *OE3* plants relative to WT plants using a significance cutoff of *P*<0.001, and a fold change (FC)>2. (**b**) Hierarchical clustering of differentially expressed genes in the transgenic lines relative to WT plants. The indicated scale is the log_2_ value of the normalized level of gene expression. (**c**) Gene ontology of biological pathways enriched in the transgenic lines based on the upregulated or downregulated genes. (**d**) qRT–PCR verification of increased expression of genes involved in plant drought response and tolerance in the transgenics under normal and drought conditions. Error bars are s.d. (**e**) Frequency of MITE insertion in the *ZmNAC111* promoter in teosinte, TST and temperate (including non-stiff stalk (NSS), stiff stalk (SS) and mixed)^51^ maize inbred lines. (**f**) Nucleotide diversity at the *ZmNAC111* locus among teosinte and MITE^−^ (maize^−^) and MITE^+^ (maize^+^) inbred lines of maize. Nucleotide diversity was compared across the *ZmNAC111* locus, among the 72 MITE^+^ and 190 MITE^−^ maize inbred lines and 42 teosinte entries. ‘P' denotes the *ZmNAC111* promoter region. Nucleotide diversity (π) for teosinte (π_T_, grey), the maize inbred lines of maize^+^ (π_+_, red) and Maize^−^ (π_−_, green) was calculated using a 100-bp sliding window and 25-bp step. The Tajima's D values of different regions are shown. *P<0.05; **P<0.01; ***P<0.001.

In the next line of our study, we compared the transcriptome of the transgenic maize and WT plants under favourable and drought conditions. A total of 628 and 443 genes were found to be upregulated and downregulated by twofold in the transgenics compared with WT, respectively, under well-watered conditions (Supplementary Fig. 10; Supplementary Data 4). Under drought stress, 547 and 425 genes were twofold upregulated and downregulated in the transgenic maize relative to WT, respectively (Fig. 6a,b; Supplementary Data 5). Biological pathways responsive to abscisic acid, ethylene and abiotic stimuli were greatly enriched among these identified upregulated genes, whereas those responsive to oxidative changes and gibberellins were especially enriched among the downregulated genes (Fig. 6c). Genes responsive to abiotic and water stresses were more significantly enriched in the upregulated genes in the drought-stressed samples as compared with the untreated ones (Fig. 6c; Supplementary Fig. 10c). We hypothesize that these transcriptomic changes may contribute to the early reduction in TR, quick stomatal closure and better protection of the photosynthesis machinery of transgenic maize under drought stress. Increased expression patterns of several well-known drought-inducible genes, such as the maize homologues of *NCED3* (ref. 43), *AFP3* (ref. 44), *RAB18* (ref. 45), *RD29B* (ref. 46), *AHG1* (ref. 47), *RD17* (ref. 45), and *DREB1D* (ref. 48) were verified in the transgenic maize (Fig. 6d). Most of these genes contain copies of NAC recognition core sequence (CACG)^49^,^50^ in their promoters, suggesting that they might be direct target genes of ZmNAC111 (Supplementary Data 6).

### Evolutionary aspects of the *ZmNAC111* locus

Teosinte (*Z. mays ssp.*), a type of wild Mexican grass, is recognized as the direct progenitor of maize based on the fact that the natural cross of teosinte and maize are fertile and the availability of a wealth of genetic domestication information^8^,^9^. After domestication from teosinte, maize cultivation spread from TST to temperate regions and maize became a major crop plant providing nutritional calories for consumption by human beings. We were interested to know the presence and distribution of the 82-bp MITE insertion in the teosinte *ZmNAC111* locus. When 96 teosinte accessions were genotyped, none of them were found to carry the MITE insertion at *ZmNAC111* indicating that this insertion might have occurred after the domestication of maize (Supplementary Data 7). Moreover, among 116 TST inbred lines, 10.34% of them were MITE^+^. Whereas, among 146 temperate lines (including stiff-stalk (SS), non-stiff-stalk (NSS) and mixed origins^51^), 41.78% were MITE^+^ (Fig. 6e). Nucleotide diversity at the *ZmNAC111* locus was found to decrease from teosinte to TST and then to temperate maize (Fig. 6f). This finding suggested that with the spread of maize cultivation from TST to temperate regions, the MITE^+^ genotype was accumulated especially in temperate maize germplasm. The MITE insertion in *ZmNAC111* locus may compromise drought tolerance of temperate maize varieties, which is in agreement with the observation that temperate subpopulation was averagely more susceptible to drought than the TST subpopulation in the whole natural variation population. Thus, we propose that the selection of the MITE^−^ genotype may help to improve drought tolerance in temperate maize inbred lines.

## Discussion

In this study, we reported that the natural variation of *ZmNAC111* gene is associated with maize drought tolerance on the whole-genome scale. On the basis of our combined GWAS and transgenic approach, we propose that the causative variation at the *ZmNAC111* locus is likely an 82-bp MITE insertion in the gene promoter, which may repress the gene expression through DNA and histone hypermethylation via the RdDM pathway. Our findings highlight the likely regulatory function of a TE in maize stress response and provide important insights into the genetic basis of the natural variation in maize drought tolerance; as well as new genetic strategies for improving this trait.

We identified the 82-bp MITE insertion in a location that was 572-bp upstream of the *ZmNAC111* coding region, which was correlated with lower *ZmNAC111* expression and drought susceptibility (Fig. 2b). Bisulphite-seq and ChIP–qPCR analyses revealed that DNA and histones are hypermethylated in the *ZmNAC111* locus in the maize inbred lines carrying the MITE (Fig. 3). The repression of *ZmNAC111* expression could be reproduced when the genomic fragment containing the MITE and *ZmNAC111* was transferred into *Arabidopsis*, indicating that the underlying molecular mechanism is likely conserved across plant species. Importantly, using the *Arabidopsis* RdDM-defective mutants, we demonstrated that the MITE-mediated *ZmNAC111* repression, at least when heterologously expressed, is dependent on RdDM (Fig. 4); which is a well-characterized RNA interference-related transcriptional gene silencing mechanism in plants^32^. This modification induces and reinforces transcriptional silencing of TEs, as well as the genes that harbor or are adjacent to the TEs^26^. Recently, whole-genome DNA methylation and RdDM surveys in maize suggest that 24-nt siRNAs are much more highly associated with transposons, which tend to be close to genes rather than the heterochromatin regions^52^. Although the majority of the maize genome exists in a heterochromatic status, which is marked by H3K9me2 and H3K27me2, RdDM was only observed to be near gene-coding regions^53^. As a result, it gave rise to the formation of CHH islands predominantly near genes, rather than in the repetitive intergenic DNA regions^52^,^53^. Our findings provide a potential molecular mechanism for how the 82-bp MITE interplays with its adjacent gene so as to contribute to drought tolerance variance in the natural maize population.

TFs play important roles in the regulation of gene expression in response to abiotic stresses, and their molecular engineering is proposed as a potential strategy for the genetic improvement of stress tolerance in crops^54^,^55^. NAC proteins constitute a plant-specific superfamily whose members participate in various regulatory and developmental processes, including stress response and tolerance^42^. The typical NAC proteins share a conserved N-terminal DNA domain but vary greatly in other regions, resulting in distinct functions of different proteins. In maize, at least 116 predicted NAC members have been identified^35^. Although ZmNAC111 was classified into an identical phylogenetic clade with OsNAC10 among the annotated NAC proteins examined (Supplementary Fig. 2), ZmNAC111 and OsNAC10 only share a 48% sequence identity on the full protein level indicating both sequence similarity and diversity between them. Previous reverse genetic studies reported that increasing *OsNAC10* expression in roots improved the yield of transgenic rice under drought^36^. Expression analysis of *ZmNAC111* in leaf samples of 133 natural maize varieties indicated that *ZmNAC111* expression was positively correlated with seedling drought tolerance under moderate and severe stresses (Fig. 2). These data suggested a positive regulatory role of *ZmNAC111* in maize seedlings exposed to water stress. Moreover, transgenic studies in both *Arabidopsis* and maize demonstrated that overexpression of the *ZmNAC111* gene could improve drought tolerance of transgenic plants and modulated stomatal closure and drought-responsive gene expression. These observations support our proposal that the elevated expression of *ZmNAC111* gene contributes to maize drought tolerance and that *ZmNAC111* acts as a positive regulator of drought response in maize (Fig. 5; Supplementary Fig. 9). Comparative transcriptome analysis of the transgenic maize and WT determined that a number of genes involved in ABA-responsiveness, such as *ZmNCED3*, *ZmRAB18*, *ZmRD29B*, *ZmRD17* and *ZmPP2C*, were upregulated indicating that *ZmNAC111* might function in an ABA-dependent stress-responsive pathway (Fig. 6d). In agreement with this result, *Arabidopsis* transgenic plants were more sensitive to exogenous ABA treatment in regard to germination and stomatal closure (Supplementary Fig. 9). In transgenic maize, the leaf SC and TR were more responsive to water deficit during the decrease of SWC, from 15 to ∼2%, in the drought treatment (Fig. 5e–h). On the basis of these results, we suggest that ABA-dependent regulation was enhanced in the *ZmNAC111* transgenic plants.

It is considered that enhancing effective use of water, which implies maximal soil moisture capture reduced non-stomatal water loss and management for minimal soil evaporation, is important for drought tolerance improvement under field conditions; however, improving WUE by reducing SC and TR may diminish plant yield in fields^56^. In this research, we did not observe obvious morphological changes in transgenic plant overexpressing *ZmNAC111* in comparison with the control plants, when they grew in pots under favourable greenhouse conditions (Fig. 5a). This was supported by the comparable measurements of the leaf PS between the transgenic and WT plants (Fig. 5e). Only on drought stress, a greater WUE was revealed in transgenic maize in comparison with WT (Fig. 5h). A similar phenomenon was also observed in transgenic rice overexpressing *SNAC1* (ref. 50). The early reduction of TR was observed in transgenic maize, when the SC remained comparable between the transgenic maize and WT (SWC>20%); which was probably due to a better or quicker osmo-adjustment (Fig. 5g). In spite of the more highly reduced SC and TR in the transgenic maize as compared with WT, the leaf PS was hardly affected when SWC was ∼15% (Fig. 5e,f). These results indicated a better protection of photosynthesis machinery or maintenance of cellular oxidative status under drought stress in the transgenic maize. Thus, the improved drought tolerance and greater WUE of transgenic maize were likely attributed to enhanced ABA signalling, quicker osmo-adjustment, better cellular protection in response to drought stress, rather than the consequence of plant growth retention. These findings also suggest that efficient water usage of plants can be improved both physiologically and genetically. Nevertheless, further intensive evaluations on important agronomic traits of transgenic plants under field conditions are needed with regard to the concerns of gene application to maize production.

In addition to SNPs, TE presence/absence variations are common and widely distributed in the maize genome, which is considered as driving force for crop evolution and domestication^57^. The MITE insertion was only present in maize germplasm but not in the teosinte accessions we examined (Fig. 6e). This finding suggests that the MITE may have inserted into *ZmNAC111* locus after maize domestication from its wild ancestor. The domestication of crops from their wild ancestors may cause the loss of genes or alleles, which are responsible for tolerance to various environmental stresses. Recently, it has been reported that the deletion of the *ZmWAK* gene during maize domestication increases susceptibility of domesticated cultivars to head smut, which is a major disease in maize production^12^. Decrease in plant stress tolerance might be exaggerated if the stress pressure is not present during the selection in breeding programme, in which high yield but not stress tolerance, is the primary goal. No evident adverse effect was observed on plant normal growth and development of the *ZmNAC111* transgenic *Arabidopsis* and maize under the favourable conditions. However, further investigation is required to test if enhancement of the *ZmNAC111* expression results in additional undesired phenotypes in field conditions. In addition, the *ZmNAC111* locus was not found to be associated with 17 important agronomic traits by analysing 513 maize inbred lines^58^. Therefore, *ZmNAC111* may be a potential candidate in gene engineering, and its MITE^−^ allele could be a selection target for the genetic improvement of drought tolerance in maize. It should be noticed that in the present study, maize drought tolerance was evaluated at seedling stage in pot-cultivated plants, which limited the characterization of above-ground tissues and vegetative growth. Whether *ZmNAC111* and its MITE^−^ allele can significantly contribute to maize yield under drought in fields demands further field-based investigation. In addition, the MITE insertion seems to be especially common in temperate maize germplasm and whether this allele confers any advantage in breeding programmes aimed for temperate regions may be an interesting theme for future research.

## Methods

### *ZmNAC111*-gene association mapping

GWAS of maize drought-tolerant genes was performed by analysing a maize natural variation panel consisting of 368 inbred lines collected from TST and temperate regions^19^. Plant drought tolerance of different inbred lines was phenotypied as previously described^24^. Briefly, the natural variation panel of maize consisting of 368 maize inbred lines was planted in a cultivation pool (6 × 1.4 × 0.22 m, length × width × depth) in which 5 ton of loam were mixed with 0.25 ton of chicken manure. To phenotype the drought tolerance of each genotype, watering was withheld when the seedlings developed three true leaves. Re-watering was applied to recover the surviving plants when clear wilting difference was observed. After rehydration for 6 days, the SR of each genotype was scored. The phenotypic data were obtained from six replicated experiments. The 56,110 genomic and 525,105 transcriptomic SNPs, with minor allele frequency (MAF) ≥0.05 were used for GWAS. The standard mixed linear model was applied (TASSEL 3.1.0)^59^, in which the population structure (Q) and kinship (K) were estimated as previously described^24^. Briefly, principal components of the association panel were calculated by EIGENSTRAT^60^ using the high-quality 525,105 SNP data with MAF≥0.05 (ref. 61). The first two dimensions were used in the principal component analysis to estimate the population structure, which could explain the 11.01% of the phenotypic variation. These results were comparable to those that were calculated by STRUCTURE. The analysis was completed by the lm function in an R program. Single-marker association analysis was initially performed to filter out markers that had no relationship with the trait (*P*≥0.995). Subsequently, 1,822 SNP markers on each chromosome were chosen to estimate the kinship coefficient (K) by SPAGeDi. Markers that were in approximate linkage equilibrium with each other were determined from PLINK^62^ based on SNP pruning (window size 50, step size 50 and the LD *R*^2^ threshold is 0.2), and the number of the subset markers was 85,806. The suggestive *P* value threshold to control the genome-wide type 1 error rate was 1.17 × 10^−5^, which was considered as the significance cutoff for the association. *ZmNAC111*-based association mapping was performed within 146 temperate and 116 TST maize inbred lines, which were representative of the whole population. The *ZmNAC111* promoter (∼0.7 kb), coding regions (including introns) and 5′- and 3′-UTR sequences were amplified and sequenced. These sequences were assembled using ContigExpress in Vector NTI Advance 10 (Invitrogen) and aligned using MEGA version 5 (http://megasoftware.net/). Polymorphisms (SNPs and InDels) were identified and their association to drought tolerance was calculated again by TASSEL 3.1.0, under the standard MLM, with MAF≥0.05.

### *ZmNAC111* gene expression analysis in different inbred lines

*ZmNAC111* expression was analysed in 133 maize inbred lines. Drought treatment was applied to the soil-grown plants at the three-leaf seedling stage by withholding water. Leaf samples were collected when the relative leaf water content (RLWC) decreased from ∼98–70%, and to 58%. Total RNA of 399 samples was isolated using TRIzol reagent (Biotopped) from a minimum of three seedlings. RNA was treated with RNase-free DNase I (Takara), and single-stranded cDNA was synthesized using recombinant M-MLV reverse transcriptase (Promega). The quantification method (2^−ΔCt^)^63^ was used and the variation in expression was estimated using three biological replicates. The maize *Ubi-2* (UniProtKB/TrEMBL; ACC: Q42415) gene was used as an internal control to normalize the data. PCR conditions consisted of an initial denaturation step at 95 °C for 10 min, followed by 40 cycles at 95 °C for 15 s, 60 °C for 30 s.

### Allelic effect of *ZmNAC111* in maize segregating populations

Three F_2:3_ segregating populations (CIMBL91 × BY4944, CIMBL55 × GEMS54 and CIMBL55 × CIMBL9) were constructed. The genotype at the *ZmNAC111* locus was analysed in ∼200 individual F_2_ plants in each population. Polymorphisms in the PCR products were visualized on 2% agarose gels. Homozygous F_2_ individuals at the *ZmNAC111* locus of the tolerant allele (MITE^−^) and the sensitive allele (MITE^+^) were self-pollinated to obtain F_3_ progenies. F_3_ progenies that were homozygous at the InDel-572 locus (MITE^−/−^ or MITE^+/+^) were mixed, respectively. Two types of F_3_ plants were grown in enriched soil (soil to vermiculite in a ratio of 1:1) in plastic boxes (0.70 × 0.50 × 0.18 m, length × width × depth) and their drought tolerance was evaluated. Each box contained 90 seedlings for each type of F_3_ plants. Three independent replications were performed in a greenhouse using 16-h light/8-h dark, 28/22 °C and a room humidity of 60% to obtain the statistical data. Drought was applied to the 10-day-old plants by withholding water. When SWC decreased from 40% to near 0%, and wilting and death of the seedlings were visible, plants were re-watered to identify surviving plants. The SR of each genotype was recorded. Three replications were carried out for statistical analyses.

### McrBC-based DNA methylation assay

Genomic DNA was isolated from fresh young leaves collected from B73 and CIMBL55 maize lines before (RLWC=98%) or after drought treatment (RLWC=70%). DNA (1 μg) was digested for 16 h at 37 °C with 10 units of McrBC enzyme, a DNA methylation-sensitive enzyme (Takara), in parallel with a mock reaction; 50 ng of digested DNA was used for qPCR reactions. DNA hypermethylation was demonstrated by the lower amount of amplification products in the qPCR analysis. All results were obtained by digesting at least two biological replicates and two independent McrBC digests. qPCR was performed using the following conditions: step 1: 95 °C, 10 min; step 2: 95 °C, 15 s; 60 °C, 30 s (40 cycles). McrBC digestion at the *ZmNAC111* gene was normalized to the reference gene maize *Ubi-2* and *Actin1* and then to the undigested control. *Arabidopsis* plants were grown on Murashige and Skoog (MS) medium agar plates for 21 days before collection. *Actin8* was used as the reference gene in *Arabidopsis*^64^. Digestion levels have been inverted to represent methylation levels.

### Bisulphite analysis

Bisulphite treatment was performed on 200 ng of genomic DNA by using the EZ DNA Methylation-Gold kit (Zymo Research, Orange, CA). After bisulphite conversion, the treated DNA was amplified by PCR. Amplified fragments were cloned into the pGEM-T vector (Promega) for sequencing. At least eight clones of each genotype were sequenced.

### ChIP assay

Fresh young leaves were collected from B73 and CIMBL55 maize lines grown under normal and drought conditions as described above. Whole plants of *Arabidopsis* were collected from *35S:ZmNAC111-B73* and *35S:ZmNAC111-CIMBL55* lines grown on the MS agar plates for 21 days. ChIP assays were performed as previously described^65^. Approximately, 7 μl of anti-H3 (Abcam; ab1791) or anti-H3K9me2 (Abcam; ab1220) antibodies were used for the ChIP assays. The amount of immunoprecipitated *ZmNAC111* chromatin was determined by qPCR on different regions of the *ZmNAC111* locus. Maize *Ubi-2* and *Actin8* were used as internal controls for maize and *Arabidopsis*, respectively. The relative abundance was normalized to the amount of DNA immunoprecipitated by a *Histone 3*-specific antibody.

### Generation of transgenics in RdDM mutant backgrounds

Two *35S:gZmNAC111-B73-2*, *-23* and *35S:gZmNAC111-CIMBL55-5*, *-12* homozygous T_2_ lines were crossed with five mutants: *dcl2-1;dcl3-1;dcl4-2* (CS16391), *drm1-2;drm2-2* (CS16383), *ago4-5* (CS9927), *rdr2-2* (SALK_059661) and *suvh4-3* (Salk_105816) (http://www.arabidopsis.org). Through PCR-based genotyping analysis, at least three independent F_2_ homozygous plants for each cross were obtained and collected. Since the *DCL3* locus was heterozygous, only *35S:gZmNAC111-B73-2* and *-23* in the *dcl2-1;dcl4-2* mutant background were obtained. The ChIP assay was performed using F_3_ RdDM-mutant homozygous plants obtained from crossing the two independent transgenic *Arabidopsis* with the RdDM mutants. The F_3_ plants germinated on kanamycin-selective medium were used for further DNA methylation and ChIP analyses. *Arabidopsis* T-DNA insertion lines were obtained from the Arabidopsis Biological Resource Center.

### *Arabidopsis* drought tolerance assays

The *ZmNAC111* genomic region in the B73 and CIMBL55 inbred lines and, the coding region in B73 were amplified and inserted into the pGreen vector^66^ under the *CaMV 35S* promoter using the *Not*I and *Xho*I restriction sites. The constructed plasmid was transformed into the GV3101 *Agrobacterium tumefaciens* strain containing the pSoup helper plasmid. *Arabidopsis thaliana* ecotype *Col-0* was transformed by *Agrobacterium*-mediated transformation and independent T_2_ transgenic lines were obtained using kanamycin-based selection. *ZmNAC111* gene expression in transgenics was determined by qRT–PCR, in which *Actin8* was used as an internal control for normalization. For the drought tolerance assays, 7-day-old plants were transferred into pots containing 250 g of soil. Thirty-two-day-old plants growing under favourable water conditions were exposed to drought stress. Water was withheld from the plants for 14 days. Watering was then resumed to allow the plants to recover. Six days later, the number of surviving plants was recorded. At least 64 plants of each line were compared with empty-vector transformed (VC) plants in each test, and statistical data were based on data obtained from three independent experiments.

### ABA-sensitivity in the transgenic *Arabidopsis*

The VC and *35S:ZmNAC111* transgenic plants were grown in parallel and collected. Seeds obtained from these plants were planted on 1/2 × MS plates containing 1% sucrose and were supplemented with or without different concentrations of ABA. Plates were chilled at 4 °C in the dark for 3 days for stratification and moved to 22 °C with a 16-h light/8-h dark cycle. Germination (emergence of radicals) was scored on the third day after germination, with three replicated assays. Stomatal aperture assays were conducted as previously described^67^. Briefly, rosette leaf peels were floated in a stomatal opening solution (10 mM MES-Tris (pH 6.15), 100 μM CaCl_2_ and 10 mM KCl) for 2 h and then transferred to a solution supplemented with various concentrations of ABA (0, 0.1, 1 and 10 μM) for another 2 h. Subsequently, the abaxial surface of each leaf was applied to 3-M clear tape to peel off the epidermal layer. Stomatal apertures were imaged and measured using ImageJ software. Forty-five stomatal apertures were analysed in each experiment and the reported values represent the mean±s.d.

### Generation and analysis of the transgenic maize

The coding region of *ZmNAC111* was amplified from B73 and the sequence-confirmed PCR fragment was inserted into the pSBII vector under the control of the *Zmubi1* promoter. The pSBI plasmid was then inserted into the LBA4404 *A. tumefaciens* strain. The LBA4404 strain, with the integrated pSBIII plasmid, was then used to deliver the *Zmubi1:ZmNAC111* expression cassette into the A188 maize inbred line as described^68^. Transgenic T_0_, T_1_ and T_2_ plants were grown in a greenhouse under a 16-h-light/8-h-dark condition. Transgenic-positive and the sibling transgenic-negative (WT) plants were determined in each generation by PCR analysis for the transgene. The expression of *ZmNAC111* in transgenic plants was determined by qRT–PCR. Three independent T_2_ lines, *ZmNAC111*-*OE1*, *ZmNAC111*-*OE3* and *ZmNAC111*-*OE7*, were selected for further analyses. Transgenic positive (individually genotyped by transgene-based PCR analysis) and WT plants were planted side by side in enriched soil (soil and vermiculite in a ratio of 1:1). Drought treatment was applied to the soil-grown plants at the three-leaf seedling stage by withholding water. After ∼20 days, watering was resumed to allow plants to recover. The number of surviving plants was recorded 7 days later. At least 15 plants of each line were compared in each test and statistical analyses were based on data obtained from three independent experiments. SC, PS and TR of the T_2_ transgenic and WT plants were measured on fully expanded leaves of seedlings at the three-leaf stage using a LICOR-6400 CO_2_ gas exchange analyser (LICOR-6400, Lincoln, NE). SWC was recorded every other day after the initiation of water withholding. Statistical analysis was based on data obtained from seven seedlings for each plant line and the experiment was repeated twice.

### RNA-seq analysis of transgenic maize

For maize RNA-seq analysis, pooled tissues from three 8-day-old maize seedlings were collected from transgenic and WT plants before or after 2-h dehydration on a clean bench to conduct the RNA-seq analysis. Total RNA was isolated using TRIzol reagent (Biotopped) and RNA integrity was evaluated using a Bioanalyzer 2100 (Agilent). The 100-bp paired-end Illumina sequencing was conducted at Berry Genomics (Beijing). An average of 3 gigabases of raw data were generated for each sample. Differential gene expression was determined using Strand NGS 2.0 software. A total of 31,501 genes were identified, representing ∼79% of all the predicted genes in maize. Enrichment analysis of gene ontology of biological pathways (GOBPs) was performed using the DAVID software program^69^ (http://david.abcc.ncifcrf.gov/) to compute *P* values that indicate the significance of each GOBP being represented by the genes. GOBPs with *P*<0.01 were identified as enriched processes. qRT–PCR of selected genes that were determined to be critical to drought tolerance was performed to verify the RNA-seq data.

### Nucleotide diversity and tests for neutrality

The genomic region of *ZmNAC111* was amplified and sequenced in 42 teosinte accessions. Nucleotide diversity (π) and the Tajima's D-statistic were calculated using DnaSP version 5.0 (ref. 70).

### Phylogenetic tree construction

The full-length amino acid sequences of 55 NAC TF encoding genes identified in maize, rice, *Arabidopsis* and sorghum were aligned using the ClustalX 1.83 program with default parameters. The phylogenetic tree was constructed based on this alignment result using the neighbour-joining method in MEGA version 5 with the following parameters: Poisson correction, pairwise deletion, uniform rates and bootstrap (1,000 replicates).

### Transactivation activity assay

cDNAs of *ZmNAC111* from 10 maize inbred lines were individually cloned into the pGBKT7 vector for evaluating protein transactivation activity in the AH109 yeast strain. The cell concentration of yeast transformants was adjusted to an OD_600_ of 0.1, and then plated on various selective plates, SD/-T, SD/-T-H and SD/-T-H-A, to compare their survival. Plates were incubated at 30 °C for 2–5 days before photographing. All the primers used in this research are listed in Supplementary Data 8.

## Additional information

**Accession codes:** The RNA sequencing data for this research have been deposited in the NCBI Sequence Read Archive under accession code SRP053172.

**How to cite this article:** Mao, H. *et al.* A transposable element in a *NAC* gene is associated with drought tolerance in maize seedlings. *Nat. Commun.* 6:8326 doi: 10.1038/ncomms9326 (2015).

## Supplementary Material

Supplementary Information Supplementary Figures 1-10

Supplementary Data 1 Variations in the ZmNAC111 genomic region and their association with maize drought tolerance.

Supplementary Data 2 ZmNAC111 expression analysis in 133 maize inbred lines under well-watered, moderate, and severe drought conditions.

Supplementary Data 3 Phenotypic comparison between ZmUbi:ZmNAC111 transgenic maize and sibling transformation-negative (WT) plants in T2 generations under well-watered conditions.

Supplementary Data 4 Genes upregulated or down-regulated in the ZmUbi:ZmNAC111 transgenic maize under the well-watered condition.

Supplementary Data 5 Genes up-regulated or down-regulated in the ZmUbi:ZmNAC111 transgenic maize after the 2h dehydration treatment.

Supplementary Data 6 NACRS motifs in the promoters of up-regulated genes involved in ABA stimulus, water deprivation, and transcription regulation.

Supplementary Data 7 Teosinte accessions genotyped at ZmNAC111 locus.

Supplementary Data 8 Primers used in this research.
