# Supplementary material for: A transposable element in a NAC gene is associated with drought tolerance in maize seedlings
Source: Nat Commun. 2015 Sep 21;6:8326. doi: 10.1038/ncomms9326 (PMC4595727; doi:10.1038/ncomms9326)
Supplement: Supplementary Information — Supplementary Figures 1-10 [file ncomms9326-s1.pdf]

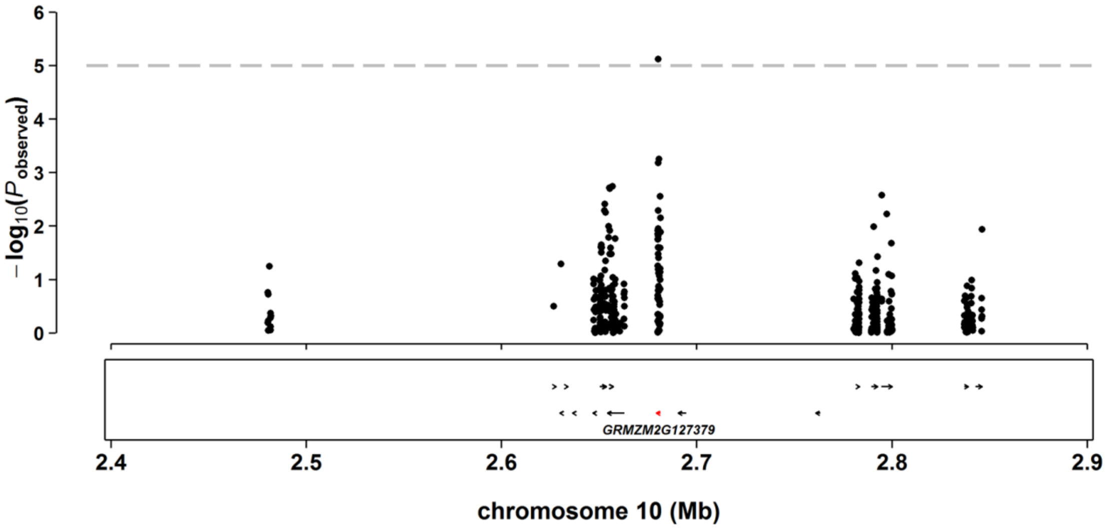

Supplementary Figure 1. Genome-wide association study analysis revealed that a SNP located in *GRMZM2G127379* was significantly associated with plant drought tolerance in maize. *GRMZM2G127379*, *ZmNAC111*, is indicated in red. A 0.5 Mb region of chromosome 10 is displayed. The physical position of the predicted genes is based on the MaizeGDB release 5b.60. The association of each marker with drought tolerance was calculated using Tassel 3.1.0, under the standard mixed linear model (MLM, MAF $\geq$ 0.05).

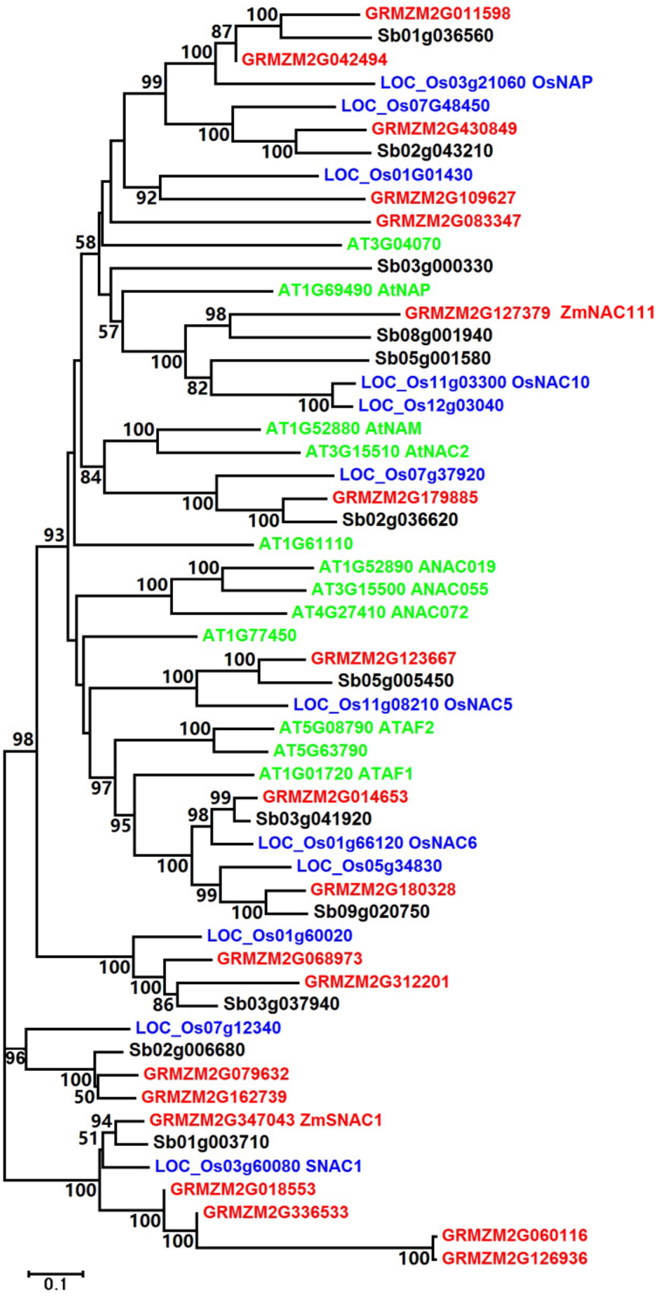

Supplementary Figure 2. Phylogenetic tree of stress-related NAC proteins in maize, rice, sorghum and *Arabidopsis*. A Neighbor-joining phylogenetic tree was constructed based on the sequence alignments of 55 full-length NAC-domain-containing proteins from four species. Gene codes and names are illustrated in red for maize; blue for rice; black for sorghum; and green for *Arabidopsis*. The bar indicates the relative divergence of the sequences examined and

bootstrap values from 1,000 replicates were displayed next to the branch.

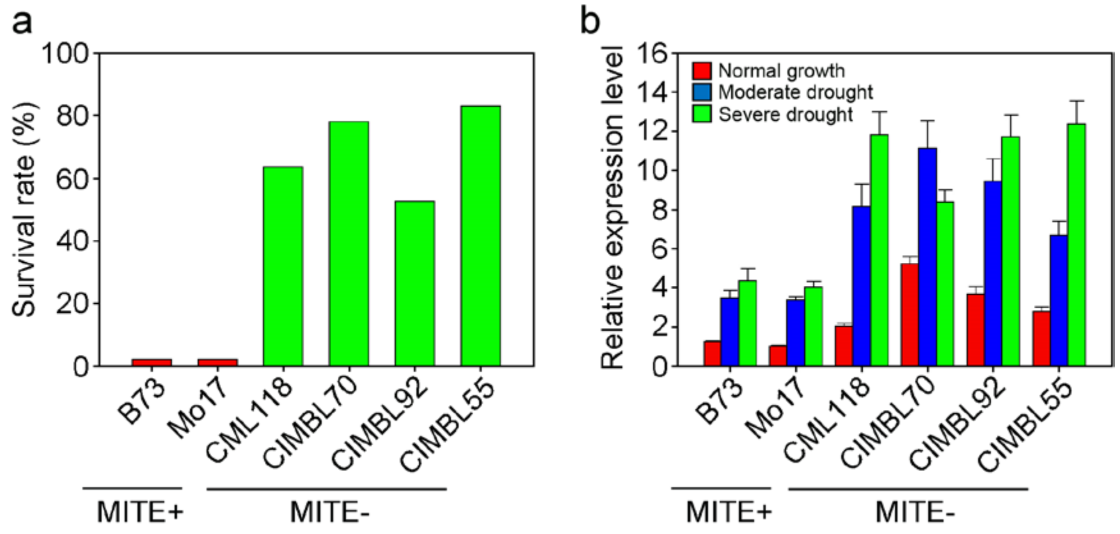

Supplementary Figure 3. Phenotype of six maize inbred lines. (a) Survival rate of B73, Mo17, CML118, CIMBL70, CIMBL92 and CIMBL55 plants subjected to severe drought stress. **(b)** Expression levels of *ZmNAC111* in B73, Mo17, CML118, CIMBL70, CIMBL92 and CIMBL55 under well-watered, moderate, and severe drought conditions. The level of drought severity was assessed as a decrease in RLWC from 98% (well-watered) to 70% (moderate drought), to 58% (severe drought). Error bars are s.d.

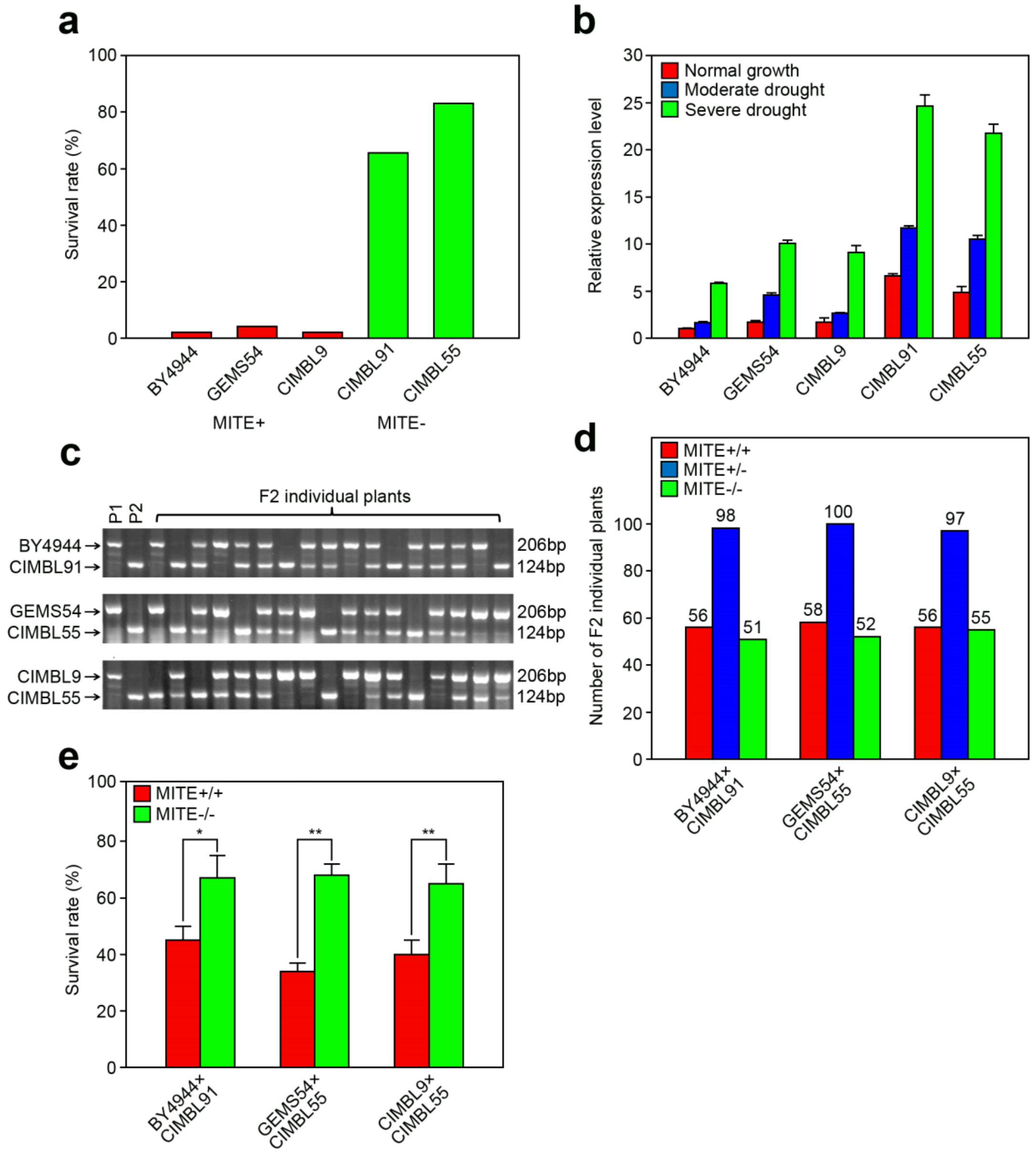

Supplementary Figure 4. The drought-tolerant allele of *ZmNAC111* co-segregates with drought tolerance in three $F_{2:3}$ populations of maize. (a) Survival rate of CIMBL55, CIMBL91, CIMBL9, GEMS54 and BY4944 plants subjected to severe drought stress. **(b)** Expression levels of *ZmNAC111* in CIMBL55, CIMBL91, CIMBL9, GEMS54 and BY4944 under well-watered, moderate, and severe drought conditions. The level of drought severity was assessed as a decrease in RLWC from 98% (well-watered) to 70% (moderate drought), to 58% (severe drought). **(c)** A representative photograph of the genotyping of F_2 individuals based on the 82-bp

MITE insertion in the three segregating populations. P1 and P2 represent the two parents of the corresponding population. The size of the DNA band from CIMBL9, GEMS54 and BY4944 was 206-bp; and the band from CIMBL55, CIMBL91 was 124-bp in length. **(d)** The number of F_2 individual plants segregating for the MITE insertion: homozygous MITE-/- (tolerant allele), homozygous of MITE+/+ (sensitive allele), and heterozygous MITE-/+. **(e)** The effect of the *ZmNAC111* tolerant allele on drought tolerance in three $F_{2:3}$ segregating populations. The survival rates of the F_3 lines carrying either the homozygous tolerant or sensitive allele of *ZmNAC111* were compared in the three populations. Error bars are s.d. and significant differences were determined using a *t*-test, * $P < 0.05$, ** $P < 0.01$.

a

Materials	Drought Tolerance	Haplotype		
		SNP1532	SNP1535	INDEL-572
B73	Sensitivity	C	A	80
Mo17	Sensitivity	C	A	80
D863F	Sensitivity	C	A	80
BY4944	Sensitivity	C	A	80
SHEN5003	Sensitivity	C	A	0
CIMBL19	Tolerance	A	G	0
CIMBL123	Tolerance	A	G	0
CIMBL22	Tolerance	A	G	0
CIMBL91	Tolerance	C	A	0
CIMBL55	Tolerance	C	A	0

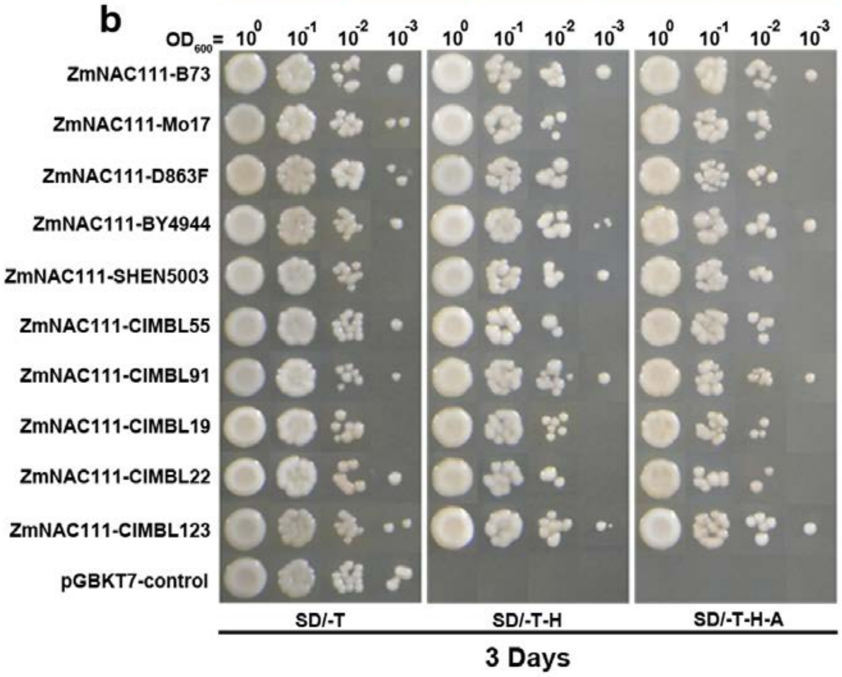

Supplementary Figure 5. Transactivation activity of different ZmNAC111 proteins encoded by the genotypes with the two non-synonymous variations. (a) The name of different maize inbred lines and their genotypes at the two significant non-synonymous sites in the coding region. **(b)** The yeast strain AH109 transformed with a vector (pGBKT7) carrying the *ZmNAC111* gene, cloned from CIMBL19, 123, 22, 91, 55, B73, Mo17, D863F, BY4944, and Shen5003 inbred lines. Cultures of transformed yeast cells were diluted and placed on agar culture plates containing a -tryptophan (-T), synthetic dropout (SD) medium (SD/-T), a -tryptophan-histidine (SD/-T-H) medium, or a -tryptophan-histidine-adenine (SD/-T-H-A) medium. The

photographs were taken of 3-day-old cultures on the corresponding medium.

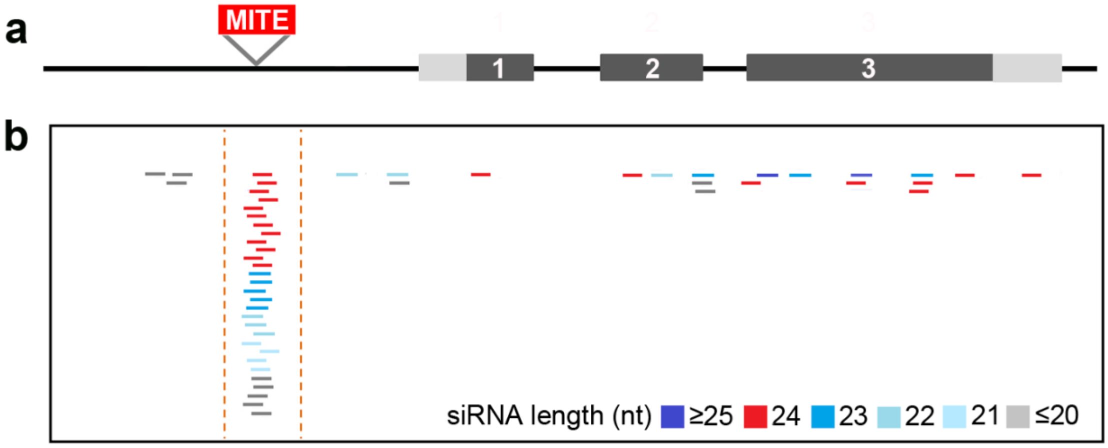

Supplementary Figure 6. The siRNAs aligned to the 82-bp MITE insertion in the *ZmNAC111-B73* allele. (a) The structure diagram of the *ZmNAC111-B73* allele. The exons are in black boxes and the MITE insertions are in a red box. **(b)** The siRNAs profiles of the *ZmNAC111-B73* allele. Tracks indicate the position of the aligned, unique siRNAs obtained from the Cereal Small RNA Database. The lengths of the mapped small-RNAs are denoted by different colors.

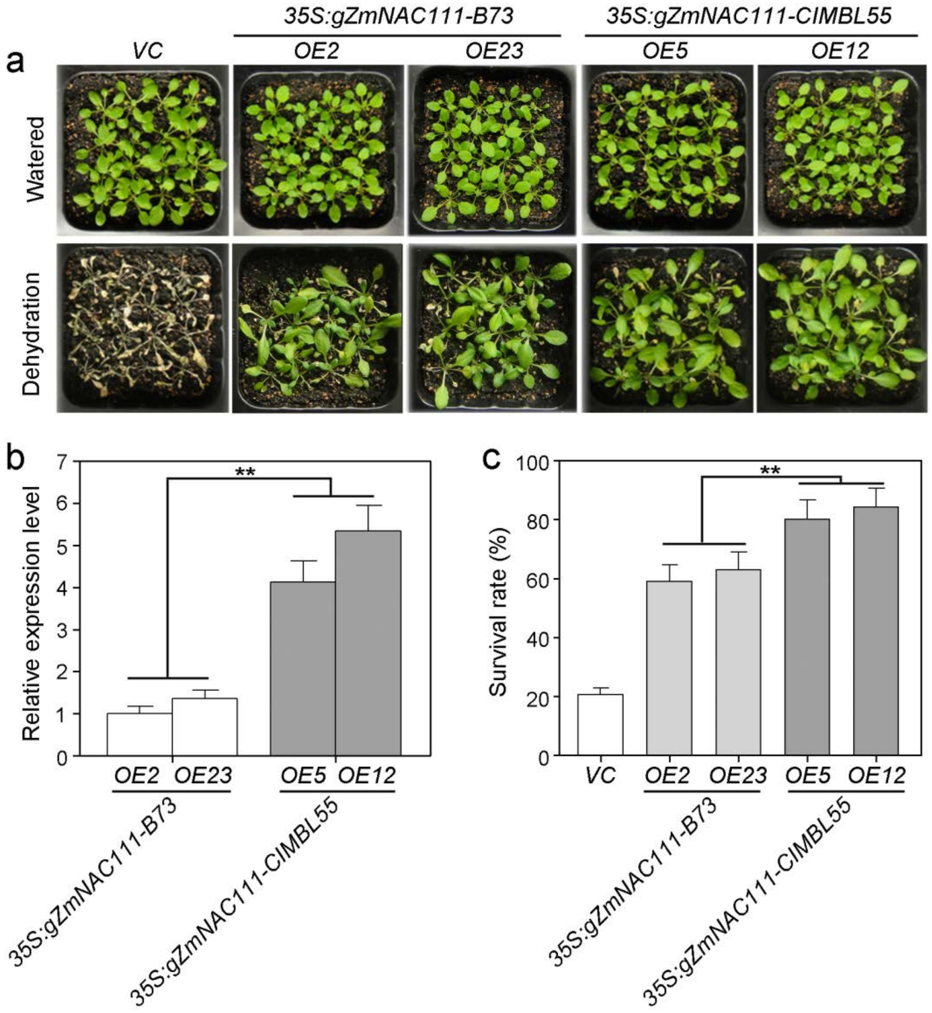

Supplementary Figure 7. Drought tolerance of *35S:gZmNAC111-B73* and *35S:gZmNAC111-CIMBL55* transgenic *Arabidopsis*. (a) Drought tolerance of *35S:gZmNAC111-B73* and *35S:gZmNAC111-CIMBL55* transgenic *Arabidopsis*. Photographs were taken before and after the drought treatment followed by a six-day period of re-watering. Vector-transformed *Arabidopsis* (*VC*) and *35S:gZmNAC111-B73-2*, *-23* and *35S:gZmNAC111-CIMBL55-5*, *-12* transgenic plants are shown. **(b)** qRT-PCR analysis of *ZmNAC111* transcript level in the four independent lines. **(c)** Statistical analysis of survival rates after the drought-stress treatment. The average survival rate and standard error were calculated based on data obtained from three independent experiments. Error bars are s.d. and significant differences were determined using a *t*-test, * $P < 0.05$, ** $P < 0.01$.

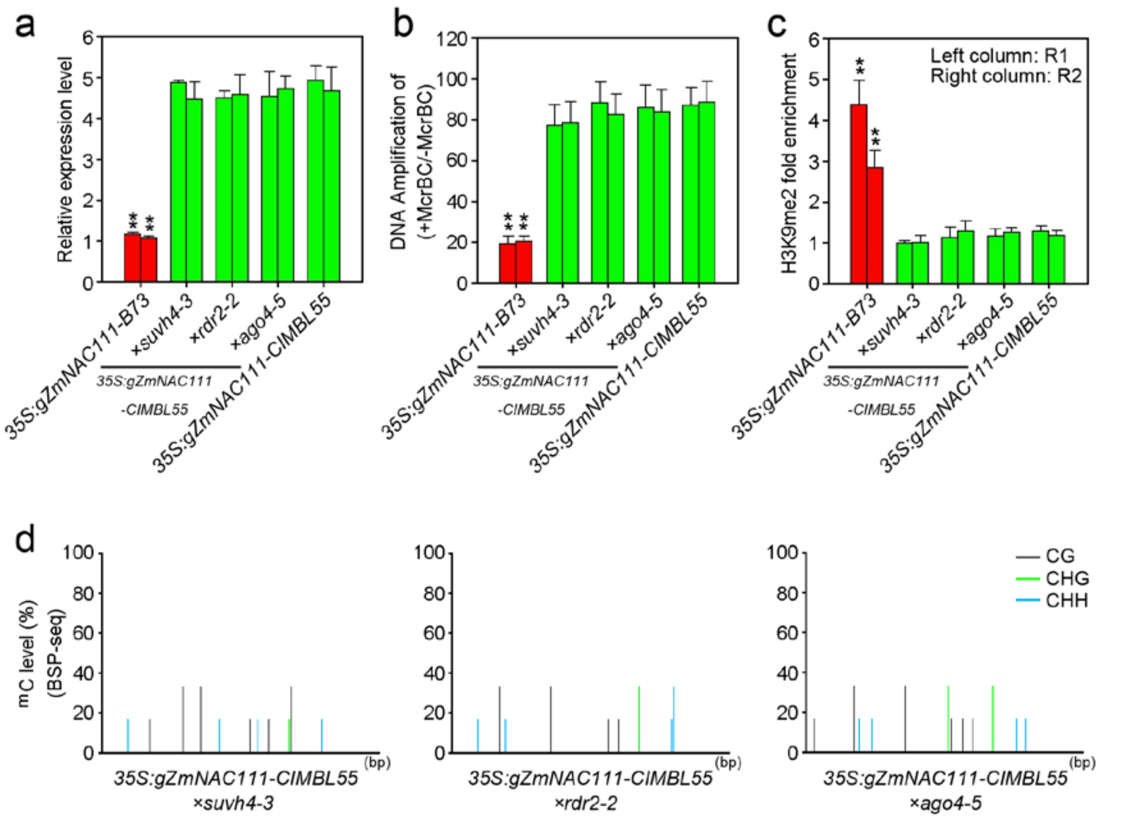

Supplementary Figure 8. DNA methylation and Histone H3K9me2 of *35S:gZmNAC111-CIMBL55* in the RdDM mutants. (a) qRT-PCR analysis of transcript levels of *35S:gZmNAC111-B73* in wild-type and *35S:gZmNAC111-CIMBL55* in wild-type and the RdDM mutant background. **(b)** DNA methylation status of the R1 region were determined by the McrBC-qPCR assay in the designated genetic backgrounds. ““×*suvh4-3*”; “×*rdr2-2*”; and “×*ago4-5*” in **(a)** and **(b)** indicate the homozygous genetic background of the *35S:gZmNAC111-CIMBL5-5* (left column) and *-12* (right column) transgenics after crossing. **(c)** Chromatin states detected by anti-H3K9me2 ChIP-qPCR assays at R1 (left column) and R2 (right column) region. The ChIP assay was performed using two independent F_3 homozygous lines in each of the designated genetic backgrounds. Green columns indicate that *ZmNAC111* expression **(a)**, DNA methylation **(b)**, and H3K9me2 **(c)** were comparable with levels in the *35S:gZmNAC111-CIMBL55* transgenics in the wild-type background; whereas red columns indicate that they were significantly different with those in the *35S:gZmNAC111-CIMBL55* transgenics. **(d)**

Methylation of cytosine residues assayed with bisulfite sequencing of the BSP1 region of the *35S:gZmNAC111-CIMBL55* transgenics in the different RdDM mutant backgrounds. Error bars are s.d. and significant differences were determined using a *t*-test, * $P < 0.05$, ** $P < 0.01$.

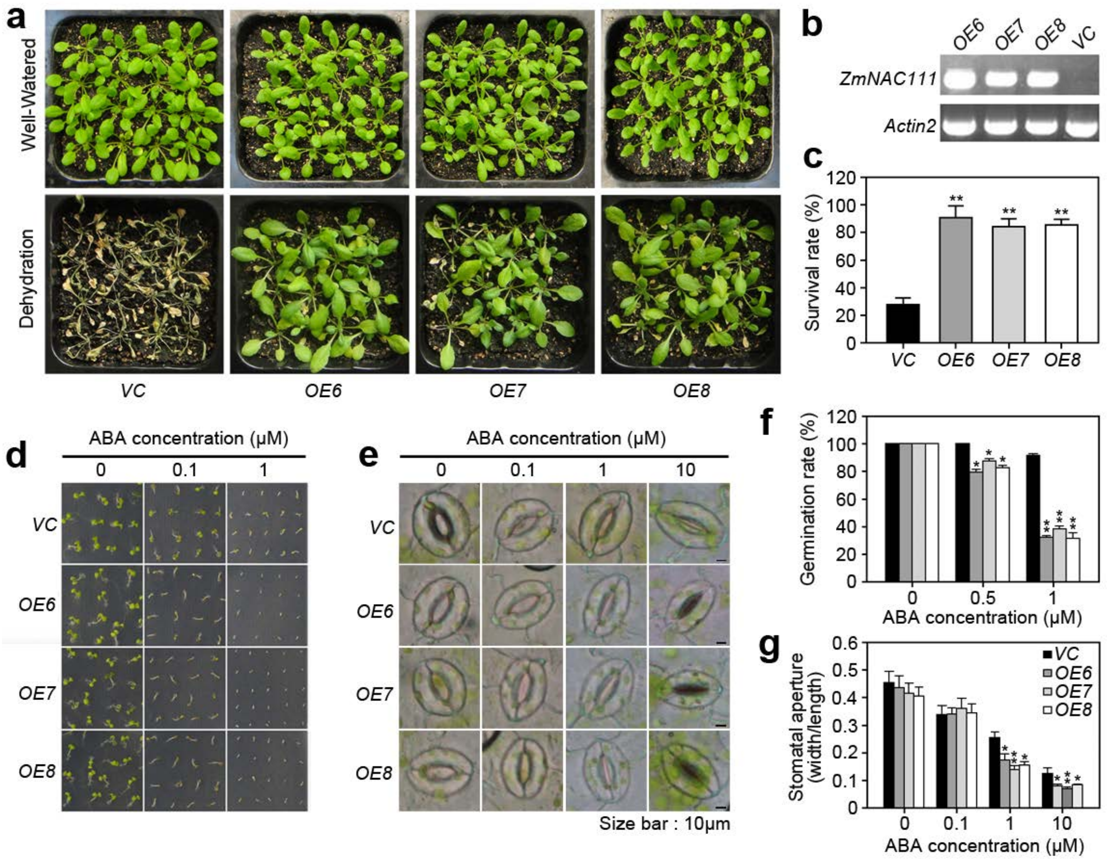

Supplementary Figure 9. Phenotype of the *35S:ZmNAC111* transgenic *Arabidopsis*. (a) Drought tolerance of transgenic *Arabidopsis* plants overexpressing *ZmNAC111*. Photographs were taken before and after the drought treatment followed by a six-day period of re-watering. Vector-transformed *Arabidopsis* (*VC*) and *ZmNAC111-OE6*, *OE7* and *OE8* transgenic plants are shown. **(b)** qRT-PCR analysis of *ZmNAC111* transcript levels in the three independent lines. **(c)** Statistical analysis of survival rates after the drought-stress treatment. The average survival rates and standard errors were calculated based on data obtained from three independent experiments. **(d)** Effect of exogenous ABA on seed germination. Seeds of *VC* and *ZmNAC111-OE6*, *OE7* and *OE8* transgenic plants were placed on half-strength MS plates supplemented with 0.5 μM and 1μM ABA and germination was scored by the appearance of radicals. Plant images were obtained 7-day after placing seeds on the MS plates. **(e)** ABA-induced stomatal closure in *VC* and *ZmNAC111-OE6*, *OE7* and *OE8* transgenic plants. Epidermal peels were used to measure the size of stomatal apertures in response to ABA at 0.1, 1.0, and 10μM. **(f)** Statistical analysis of **(d)** was

based on data obtained from three independent experiments using 100 seeds in each experiment. **(g)** Statistic analysis of **(e)** was based on data obtained from three replicates and the presented values represent the means ± s.d. (n=45). Error bars are s.d. and significant differences were determined using a *t*-test, * $P < 0.05$, ** $P < 0.01$.

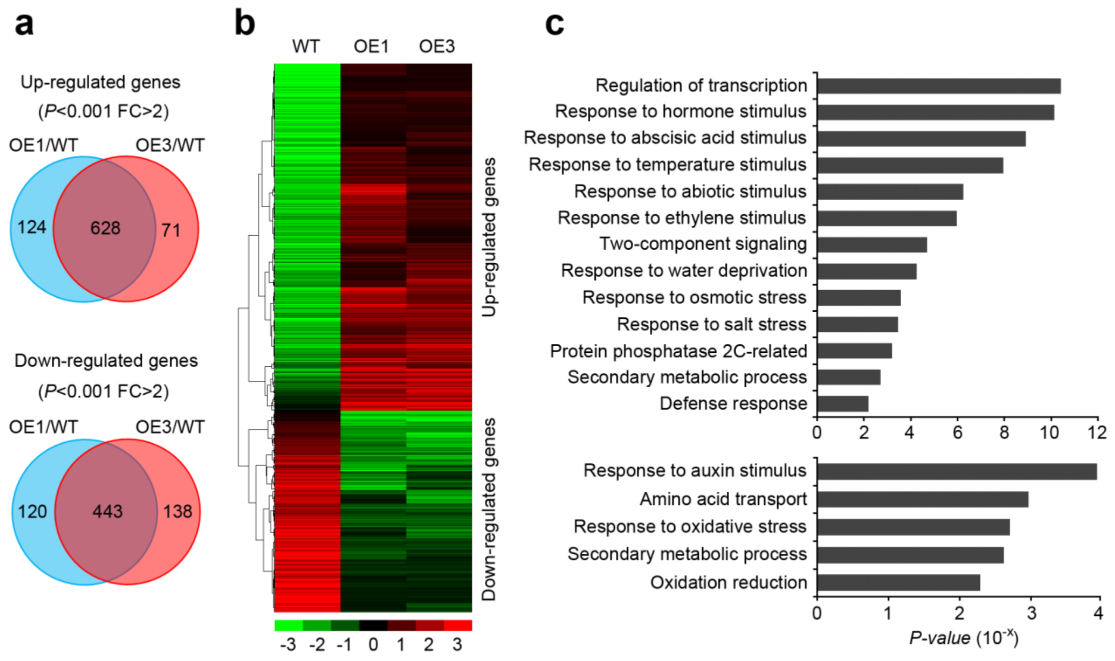

Supplementary Figure 10. Transcriptomic analysis of *ZmUbi:ZmNAC111* transgenic maize under well-watered conditions. (a) Venn diagrams of *ZmNAC111* up-regulated and down-regulated genes (P < 0.001, FC > 2.0) in two *ZmUbi:ZmNAC111-OE1 and ZmUbi:ZmNAC111-OE3* transgenic maize in relation to *WT* plants (*OE1* and *OE3*). **(b)** Hierarchical clustering of *ZmNAC111* up-regulated and down-regulated genes in *OE1* and *OE3* plants. The scale represents the $\log_2$ value of the normalized level of gene expression. **(c)** Enriched GOBPs based on up- and down-regulated genes (P < 0.01, P-value was computed by DAVID, indicating the significant of the enrichment) in *ZmNAC111* transgenic plants.
